# Excellent Response to Oral Isotretinoin in a Pediatric Patient With Dissecting Cellulitis of the Scalp

**DOI:** 10.7759/cureus.109194

**Published:** 2026-05-19

**Authors:** Nehaa Sohail, Mary Cavanagh, Ayaan Sohail, Brent Kelly, Ayezel Munoz Gonzalez

**Affiliations:** 1 Medical Education, Texas Tech University Health Sciences Center Paul L. Foster School of Medicine, El Paso, USA; 2 Dermatology, University of Texas Medical Branch, Galveston, USA; 3 Medicine, John P. and Kathrine G. McGovern Medical School, The University of Texas Health Science Center, Houston, USA

**Keywords:** dissecting cellulitis, isotretinoin, scalp, therapy, treatment

## Abstract

Dissecting cellulitis of the scalp (DCS) is a rare, chronic inflammatory scalp disorder that is characterized by painful, recurrent nodules, abscesses, fistulas, and sinus tracts that can lead to scarring alopecia. We present the case of an 11-year-old Black female with painful, pruritic scalp nodules and hair loss of several months' duration. The patient's clinical and histopathologic presentation was consistent with DCS. After failure to improve with numerous oral/topical antibiotics and topical/intra-lesional corticosteroid therapies, the patient achieved an excellent clinical response to oral isotretinoin. The treatment of DCS remains a challenge for dermatologists due to the lack of standardized, evidence-based guidelines. Given the complexity of treating DCS, especially in the pediatric population, raising awareness for effective management options may lead to enhanced patient outcomes.

## Introduction

Dissecting cellulitis of the scalp (DCS) is a rare, chronic inflammatory scalp disorder characterized by painful, recurrent nodules, abscesses, fistulas, and sinus tracts that can lead to scarring alopecia [1]. DCS is part of the follicular occlusion tetrad, which also includes acne conglobata, hidradenitis suppurativa, and pilonidal cysts, sharing a common pathogenesis related to follicular hyperkeratosis and subsequent inflammation [2]. The exact etiology of DCS remains unclear, but it is believed to involve a combination of genetic predisposition, immune dysregulation, and microbial involvement. The treatment of DCS remains a challenge for dermatologists due to the lack of standardized, evidence-based guidelines.

## Case presentation

An 11-year-old Black female with no significant past medical history presented in March 2022 with a five-month history of scalp bumps, hair loss, pain, and pruritus. The condition initially began as a solitary "boil" on the scalp that drained spontaneously and was subsequently followed by progressive hair loss. There were no clear inciting triggers identified besides the onset of puberty. During this time, the patient opted to wear her hair naturally with no protective heat styles, such as box braids, twists, or wigs. Prior to presentation, she had been treated with a 10-day course of cephalexin and oral griseofulvin prescribed by an outside provider without improvement.

**Figure 1:**
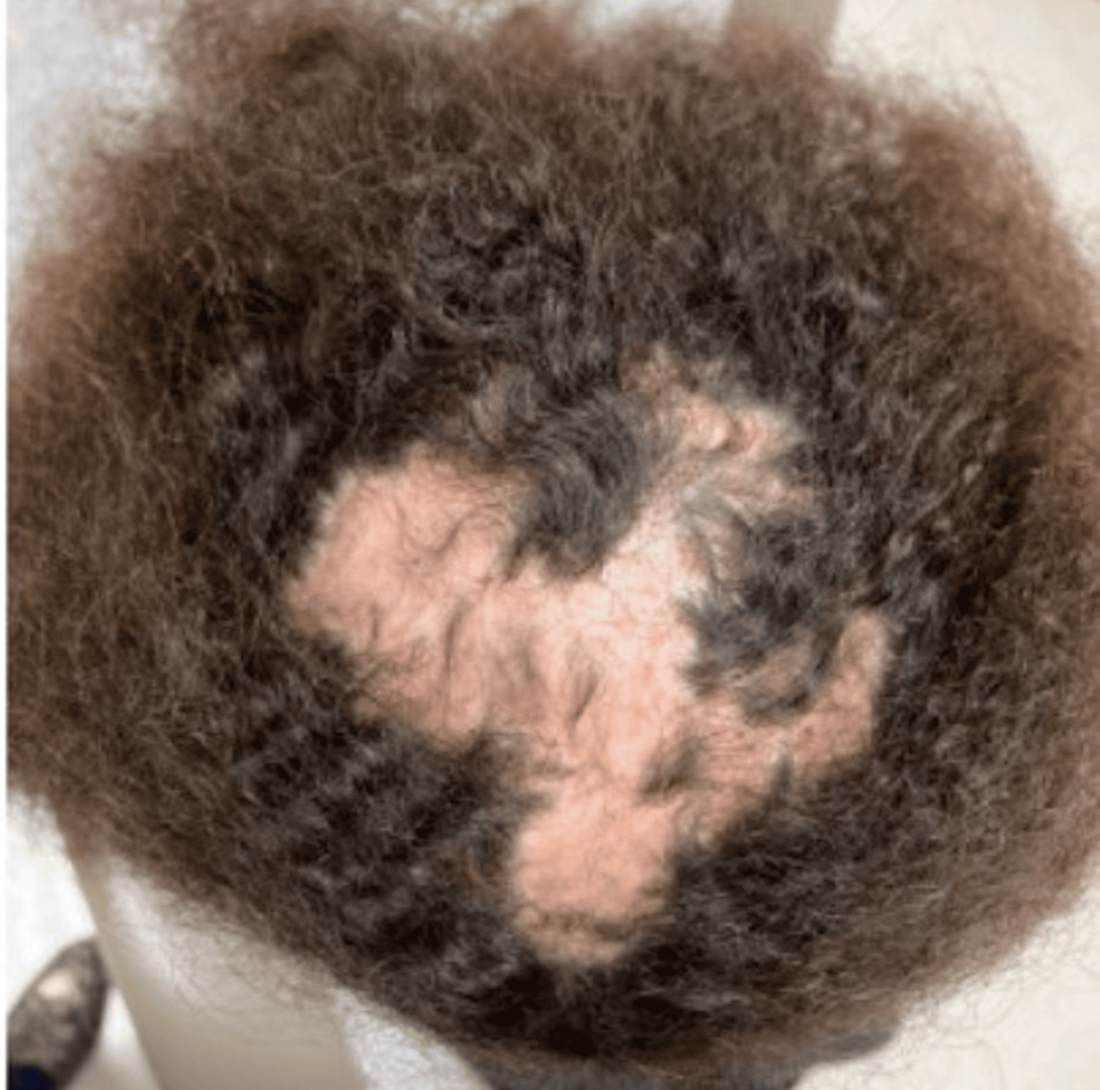
Initial presentation of scalp demonstrating ill-defined patches of boggy cicatricial alopecia

On initial examination, the scalp demonstrated ill-defined patches of boggy cicatricial alopecia (Figure 1). A punch biopsy was obtained along with triple tissue cultures, including bacterial, fungal, and acid-fast bacilli cultures, all of which were negative. The differential diagnosis included tinea capitis, folliculitis decalvans, discoid lupus erythematosus, pilar cysts, pseudopelade of Brocq, and acne keloidalis nuchae.

**Figure 2:**
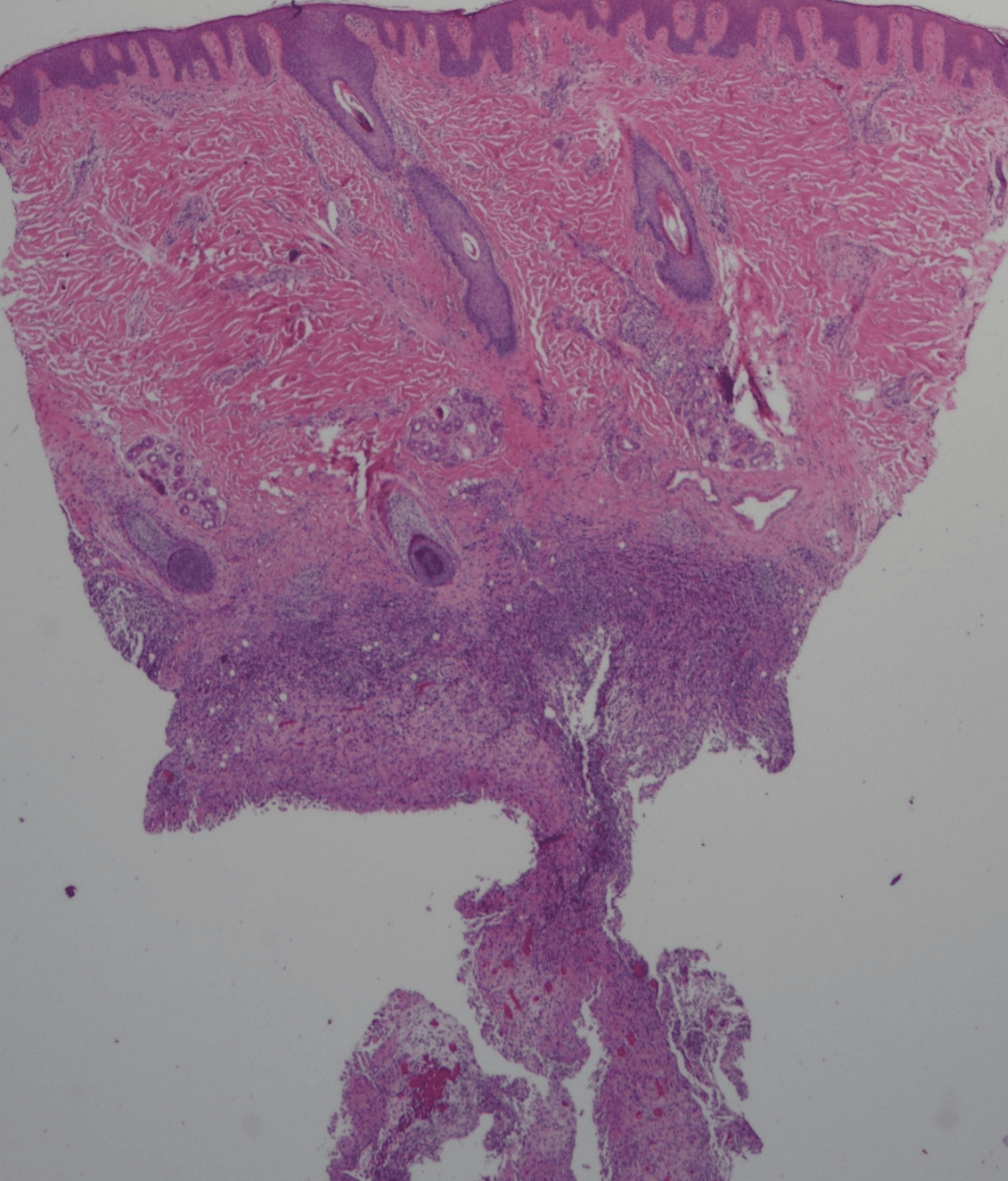
Histopathologic examination (20x) demonstrating deep dermal chronic, granulomatous inflammation with granulation tissue, most consistent with DCS DCS: Dissecting cellulitis of the scalp

**Figure 3:**
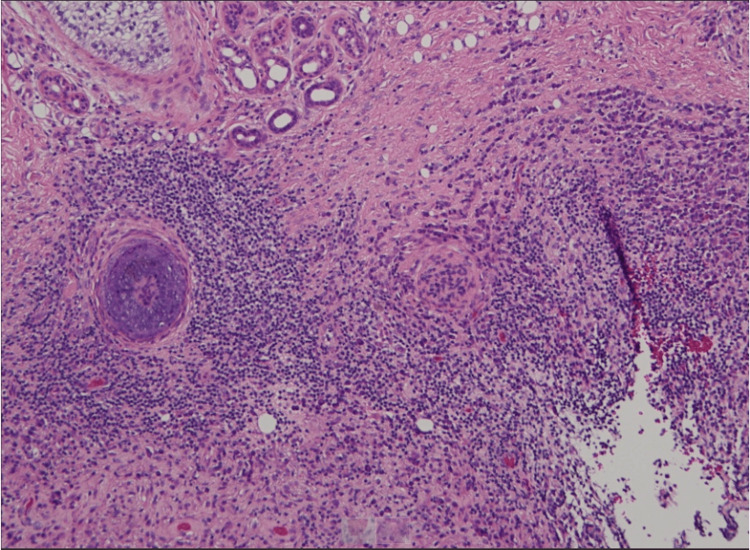
Histopathologic examination (100x) demonstrating deep dermal chronic, granulomatous inflammation with granulation tissue, most consistent with DCS DCS: Dissecting cellulitis of the scalp

However, histopathologic examination revealed deep dermal chronic granulomatous inflammation with granulation tissue, most consistent with DCS (Figures 2-3).

**Figure 4:**
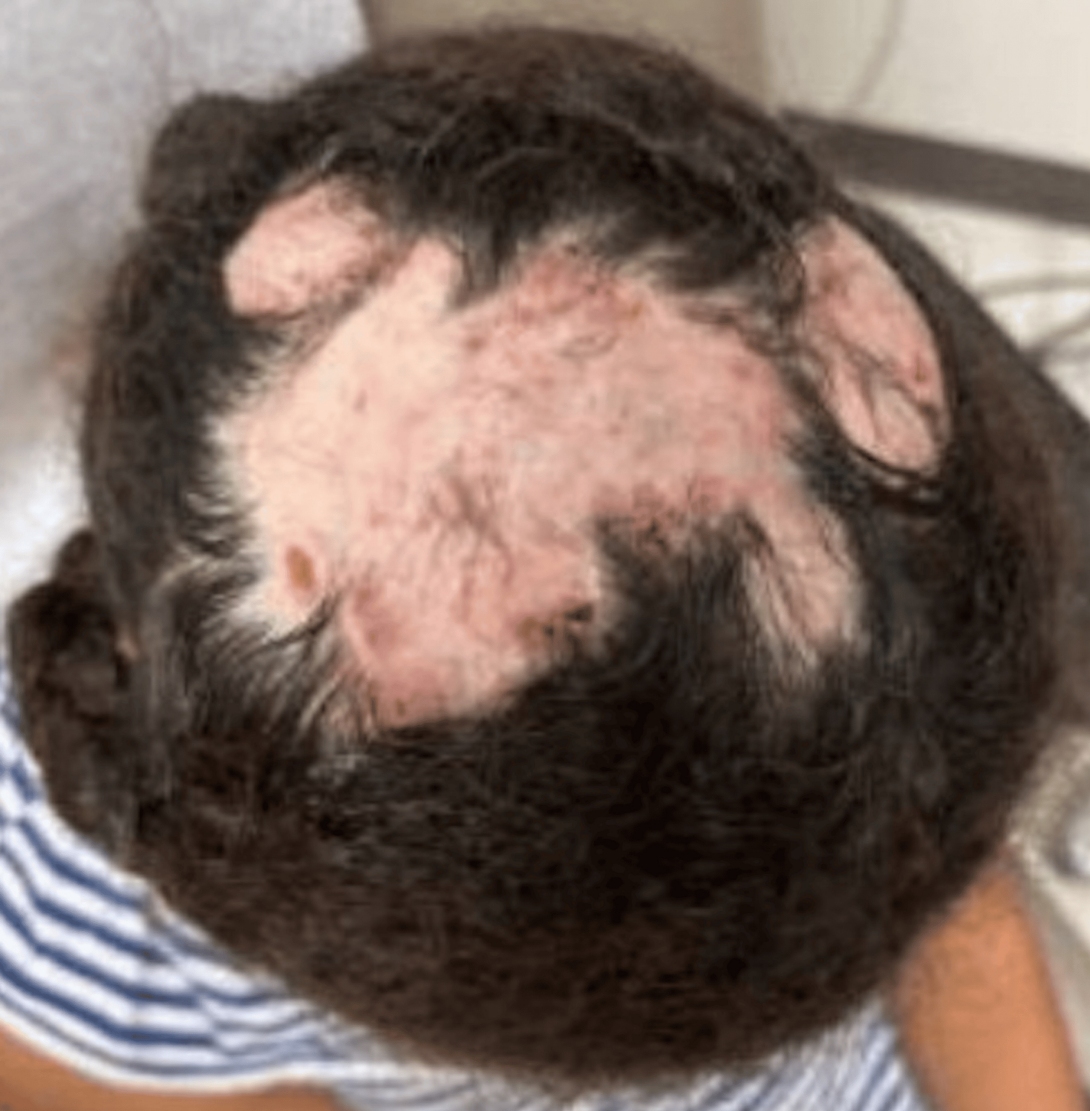
Subsequent presentation demonstrating worsening cicatricial alopecia with scattered erosions and crusted papules

The patient was initially treated with oral doxycycline 100 mg twice daily and topical clobetasol 0.05% solution twice daily. By June 2022, the patient demonstrated mild improvement, and intralesional triamcinolone (ILK) at 5 mg/mL was administered to the affected areas. Despite continued therapy, disease activity persisted, which prompted a two-week course of oral terbinafine in July 2022. This was due to concern for occult tinea capitis given worsening alopecia (Figure 4).

In August 2022, the patient showed some improvement with early hair regrowth; however, intermittent flares continued. In October 2022, she developed a new tender draining nodule requiring incision and drainage with negative wound cultures. Over the following months, she experienced fluctuating disease activity despite continued doxycycline, topical clobetasol, and serial ILK injections (2.5-10 mg/mL).

Given the chronic, relapsing course, systemic therapies were discussed extensively. Although both adalimumab and isotretinoin were considered, the patient's parents initially declined these options. In March 2023, due to persistent recalcitrant disease, doxycycline was discontinued, and a combination regimen of oral clindamycin 300 mg twice daily and rifampin 300 mg twice daily was initiated. This treatment resulted in partial improvement.

Ultimately, after further counseling, the patient was initiated on oral isotretinoin in May 2023 following iPledge registration. Concurrent clindamycin and rifampin were discontinued, and adjunctive therapy with topical clindamycin gel, benzoyl peroxide wash, and intralesional corticosteroids was continued. The patient completed an eight-month course of isotretinoin dosed at 1 mg/kg/day, achieving a cumulative dose of 227.5 mg/kg.

**Figure 5:**
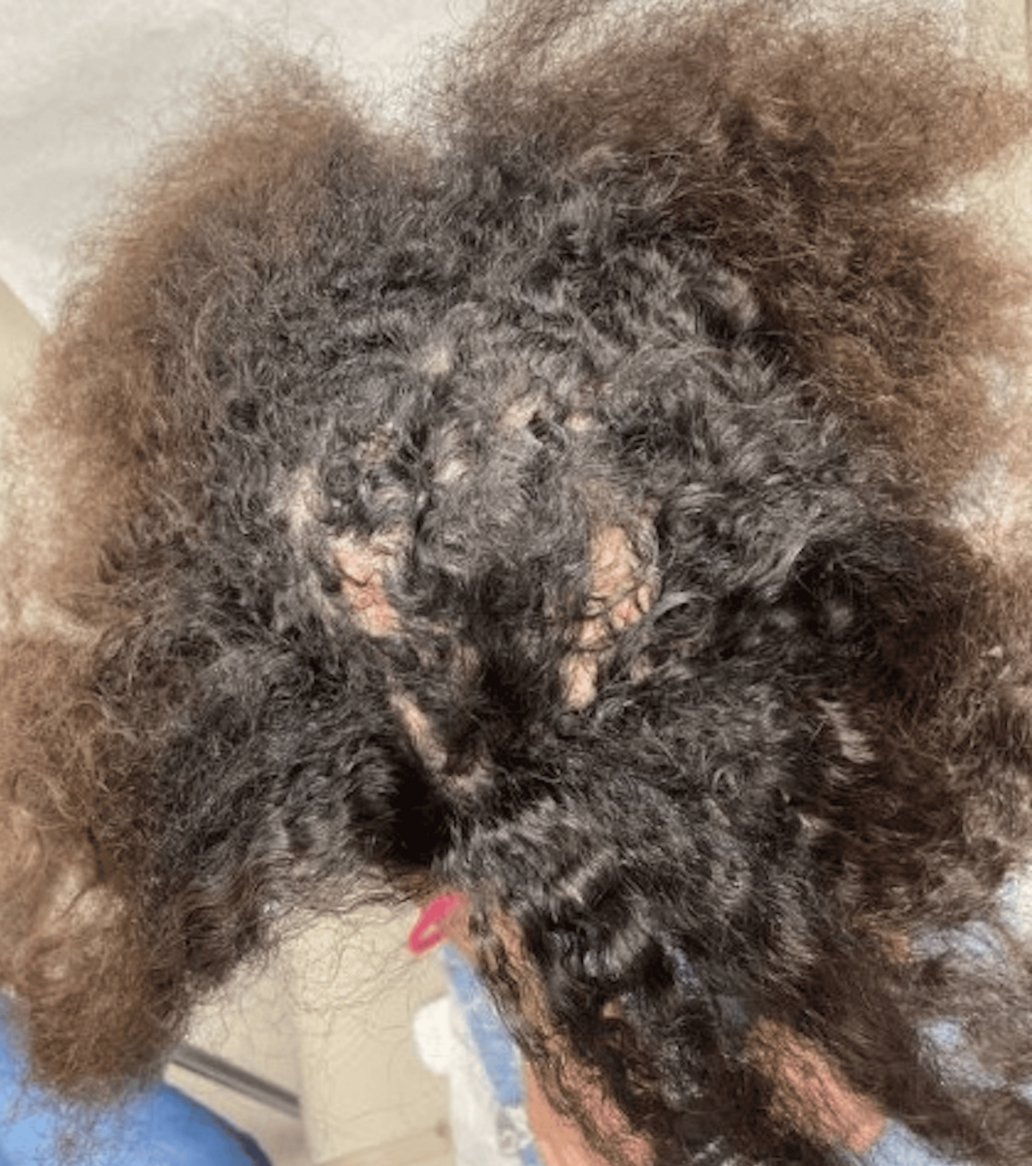
Following a course of oral isotretinoin, significant hair regrowth was noted with remaining small, focal alopecia patches

Following completion of isotretinoin therapy in January 2024, the patient demonstrated significant clinical improvement with substantial hair regrowth and only small residual focal patches of alopecia (Figure 5). Maintenance therapy included topical corticosteroids and topical clindamycin. Although oral dapsone was considered for long-term disease control, it was declined by the patient's family.

At the most recent follow-up in January 2026, the patient's DCS remained stable with no additional flares and is currently managed with intermittent use of topical fluocinolone oil (Derma-Smoothe) as needed.

## Discussion

This case seeks to highlight a rare presentation of DCS in a Black, female child, as well as the impressive treatment response to oral isotretinoin at a dose of 1 mg/kg/day. Conventional treatments for DCS include systemic antibiotics, corticosteroids, and surgical intervention. Antibiotics, such as clindamycin, rifampicin, and tetracyclines, are commonly used for their anti-inflammatory and antimicrobial properties, yet they often provide limited or temporary relief. Oral retinoids have been the most extensively studied medical and procedural treatments for DCS [1]. Corticosteroids may help reduce inflammation, but prolonged use in pediatric patients carries risks of growth suppression, immunosuppression, and metabolic side effects. Surgical options, such as drainage or excision, may address abscesses or sinus tracts but are not curative and can lead to additional scarring. Given these limitations, finding effective and safe treatment modalities for DCS is crucial in pediatric cases.

Isotretinoin has shown efficacy in managing DCS due to its multifaceted action [3,4]. It works by inhibiting sebaceous gland function, reducing sebum production, and thereby limiting the follicular environment that fosters bacterial proliferation. Additionally, isotretinoin modulates keratinocyte differentiation, preventing follicular hyperkeratinization, which is thought to play a central role in the pathogenesis of DCS [4]. This reduction in follicular occlusion and sebaceous activity reduces inflammatory infiltrates, halts disease progression, and allows for healing of sinus tracts and abscesses. Otherwise, the risks of isotretinoin use in children are comparable to those in adults.

In this case, oral isotretinoin led to a marked clinical improvement with a reduction in inflammatory lesions, sinus tracts, and nodules. This positive outcome aligns with prior case reports and studies demonstrating isotretinoin's efficacy in DCS, although literature describing its use in pediatric populations remains limited. DCS is often chronic and relapsing despite treatment. However, a meta-analysis demonstrated an overall isotretinoin efficacy rate of approximately 90%, with recurrence occurring in roughly 24% of patients following treatment response [4]. Therefore, the durable improvement observed in our patient after a documented cumulative isotretinoin course and long-term follow-up is clinically meaningful. This case further supports isotretinoin as an effective systemic therapy for DCS while emphasizing the importance of adequate cumulative dosing, close follow-up, and early intervention to reduce the risk of permanent scarring alopecia and hair loss.

Isotretinoin, while effective, requires close monitoring, particularly in pediatric patients. Rare reports of premature epiphyseal closure have been seen with standard acne dosing of isotretinoin (0.5-1 mg/kg/day) in pediatric patients; however, premature epiphyseal closure occurs more commonly with use of higher isotretinoin doses for treatment of neuroblastoma [5-10]. More concerning are the risks of teratogenicity, hepatotoxicity, and dyslipidemia, necessitating regular liver function tests and lipid profiles during therapy. For female patients, counseling on contraceptive options and strict pregnancy prevention programs are standard in adolescents and may present a challenge in younger populations. In this case, regular monitoring allowed for early detection and management of mild side effects, facilitating uninterrupted treatment and an excellent response.

## Conclusions

Given the chronic and relapsing nature of DCS, this case adds valuable insight into achieving long-term remission in pediatric patients, ultimately improving their quality of life. Early intervention can prevent irreversible scarring alopecia and psychosocial impact, which emphasizes the need for prompt diagnosis and consideration of systemic therapies in severe pediatric cases of DCS. The treatment of DCS remains a challenge for dermatologists due to the lack of standardized, evidence-based guidelines. Furthermore, DCS is often refractory to treatment. Improved awareness of the presentation and treatment options for DCS in pediatric patients may lead to earlier detection and more effective management, ultimately enhancing patient outcomes.
